# Localization of FGF21 Protein and Lipid Metabolism-Related Genes in Camels

**DOI:** 10.3390/life13020432

**Published:** 2023-02-03

**Authors:** Yuan Gao, Shuqin Zhao, Wangdong Zhang, Huaping Tang, Meilin Yan, Fang Yong, Xu Bai, Xiaochun Wu, Yong Zhang, Quanwei Zhang

**Affiliations:** 1 College of Life Science and Technology, Gansu Agricultural University, Lanzhou 730070, China; 2 Gansu Key Laboratory of Animal Generational Physiology and Reproductive Regulation, Lanzhou 730070, China

**Keywords:** FGF21, hormone, immunohistochemistry, fat metabolism

## Abstract

With the ability to survive under drought and chronic hunger, camels display a unique regulation characteristic of lipid metabolism. Fibroblast growth factor (FGF) 21 is a peptide hormone that regulates metabolic pathways, especially lipid metabolism, which was considered as a promising therapeutic target for metabolic diseases. To understand the FGF21 expression pattern and its potential relationship with lipid metabolism in camels, this study investigated the distribution and expression of FGF21, receptor FGFR1, and two lipid metabolism markers, leptin and hormone-sensitive lipase (HSL), using an immunohistochemistry (IHC) assay. The results showed that FGF21 was widely expressed in camel central nerve tissue and peripheral organs but absent in lung and gametogenic tissue, including the testis, epididymis, and ovary. In striated muscle, FGF21 is only present at the fiber junction. FGFR1 is expressed in almost all tissues and cells, indicating that all tissues are responsive to FGF21 and other FGF-mediated signals. Leptin and HSL are mainly located in metabolic and energy-consuming organs. In the CNS, leptin and HSL showed a similar expression pattern with FGFR1. In addition, leptin expression is extremely high in the bronchial epithelium, which may be due to its role in the immune responses of respiratory mucosa, in addition to fat stores and energy balance. This study found that FGF21 showed active expression in the nervous system of camels, which may be related to the adaptability of camels to arid environments and the specific regulation of lipid metabolism. This study showed a special FGF21-mediated fat conversion pattern in camels and provides a reference for developing a potential therapeutic method for fat metabolism disease.

## 1. Introduction

Camels belong to the Camelidae family, order *Artiodactyla*, sub-order *tylopoda*. The Camelidae family comprise two main types, large and small camelids. The small camelids originate from the Andin Mountains of South America. The large camelids are represented by two domesticated species, the one-humped camel (dromedary) and the two-humped camel (Bactrian) [1]. Camels are multipurpose animals. They can be used for milk, meat and wool production, for transportation, racing contests, tourism, and agricultural work [1,2]. The camel is a domesticated ruminant species of considerable biological and economic importance, especially in arid and semi-arid regions. To survive in these difficult conditions, the camel has developed exceptional physiological and biochemical particularities, especially the adaption mechanisms of nutrition (urea recycling), energy (fat reserves of the hump), and water (resistance to thirst) [3,4]. Intriguingly, camels are able to survive long periods without feeding due to their efficient lipid reserves in their hump. However, changes in their lipid reserves and related mechanisms are not currently well-understood.

The fibroblast growth factor (FGF) 21 gene was cloned in 2000, and the first insights into the biological significance of this protein were in 2005 [5]. Unlike the canonical growth-stimulating FGFs that classically function in mitosis, differentiation, and angiogenesis, FGF21, together with FGF19 and FGF23, belong to a hormone-like subgroup within the FGF superfamily, are characterized by their reduced binding affinity for heparin that enables them to be transported in the circulation, function in an endocrine manner, and act as a potent regulator of glucose and lipid metabolism but was not observed to be mitogenic in vivo [5,6]. FGF21 signals via FGF receptors. In contrast to the majority of FGFs, FGF21 requires a cofactor, β-Klotho (KLB), to bind the FGF receptors and activate the downstream cascade in tissues [6,7,8]. 

FGF21 is a hormone that regulates important metabolic pathways. FGF21 stimulates the oxidation of fatty acids, production of ketone bodies, inhibition of lipogenesis, glucose uptake, amino acid transport, and energy expenditure [9,10,11]. The finding that FGF21 regulates glucose–lipid metabolism has made it a promising therapeutic target for metabolic disease. Actually, serum FGF21 is strongly associated with liver fat content and fat accumulation, making FGF21 a valuable diagnostic marker [12,13,14]. There is evidence that administering FGF21 can induce sustained weight loss, ameliorate hepatic steatosis, lower blood glucose and triglyceride (TG) levels, improve insulin sensitivity, increase brown adipocyte numbers, and preserve β-cell function and mass [15,16,17]. FGF21 is expressed in several metabolically active organs, such as the liver, adipocytes, pancreas, brain, testes, muscle, and heart, but FGF21 regulation is complicated because of its different tissue production and action [16,18,19]. 

Obesity is constantly increasing worldwide due to the progressive globalization of sedentary lifestyle and diets rich in lipids and processed food, which increases the risk of many diseases, including cancers and cardiovascular and immune-mediated conditions [20,21]. The study of camel fat metabolism would be helpful to understand more about the fat conversion mode, which will provide a reference for developing a potential therapeutic method for human obesity. 

## 2. Materials and Methods

### 2.1. Camels and Tissues

Healthy Alashan Bactrian camels at 6–8 years with 510 kg of male camels and 450 kg of female camels were obtained from the slaughterhouse (Zhangye, Gansu province of China) and were anaesthetized intravenously with sodium pentobarbital (20 mg/kg) and then exsanguinated to death. Fresh tissues samples after collection and fixation in 10% neutral formalin solution were assigned to laboratory for microsection.

### 2.2. Antibodies

Primary antibodies anti-FGF21 (ab171941, 1:300 dilution), anti-FGFR1 (ab10646, 1:200 dilution), anti-leptin (ab3583, 1:200 dilution), and anti-HSL (ab220074, 1:200 dilution) were obtained from Abcam (UK). Secondary antibodies, including SABC anti-rabbit and anti-mouse IgG (1:500 dilution), were purchased from Bioss (Beijing, China).

### 2.3. Microsection

The paraffin sections were made and stained with hematoxylin and eosin (H&E) using a routine method [22] and SABC-immunohistochemistry (IHC) using the method described as follows: the samples were sectioned (4 μm) and placed on the polylysine-coated slides (molecular weight: 150,000–300,000; concentration: 0.10% (*w*/*v*) in water, Sigma, St. Louis, MO, USA). After deparaffination, enzyme-induced epitope retrieval was performed using 1.0 mg/mL trypsin 1:250 (250.N.F.U/mg, Sigma, St. Louis, MO, USA) treatment, which was followed by endogenous peroxidase blocking (3% H_2_O_2_). Then, blocking was performed using 5% bovine serum albumin (BSA, Boster, Wuhan, China) treatment. All samples with the primary antibody were incubated at 4 °C overnight. After being rinsed with PBS 5 min × 3 times, HRP-conjugated secondary antibody was applied for 1 h in humidified box at 37 °C. After being rinsed with PBS 5 min × 4 times, the SABC was applied for 30 min in humidified box at 37 °C. After being rinsed with PBS 5 min × 4 times, DAB Kit (ZSGB-BIO, Beijing, China) was used at room temperature for detection. Slides were counterstained with hematoxylin (Solarbio, Beijing, China) and mounted with neutral balsam (Solarbio, Beijing, China). Sections were examined using an Olympus microscope (Olympus, Hamburg, Germany).

### 2.4. Light Microscopy

The location and expression sites of each protein were observed and photomicrographed using DP-71 microscopy system (Olympus, Tokyo, Japan).

### 2.5. Statistical Analysis

Three sections and six microscopic fields were randomly selected for each sample. The mean optical density (OD) of each site was calculated using Image-Pro Plus 6.0 (Media Cybernetics Co., Rockville, MD, USA). The detection was performed as described previously [22]. All data are presented as the mean ± standard deviation (SD) of repeated experiments. The differences among groups were analyzed using two-tailed Student’s *t*-test. *p*-values of <0.05 were considered to be significant.

## 3. Results

FGF21 is a key hormone that regulates metabolic pathways, especially in lipid metabolism [9] via a receptor FGFR1-dependent manner [23,24]. To understand FGF21 and its receptor FGFR1 expression patten, we found which tissue and cells produced the FGF21 factor and explored the correlation between FGF21 and lipid metabolism in camels. Immunohistochemistry (IHC) was performed using anti-FGF21 antibodies, anti-FGFR1 antibodies, and some lipid metabolism markers, including leptin and hormone-sensitive lipase (HSL) antibodies, to analyze these proteins’ distribution in various camel tissues, including the central nervous system (CNS), reproductive system, adipose tissue, muscle, and visceral organs. Histochemical staining data are shown as follows: 

### 3.1. In the CNS

**Figure 1:**
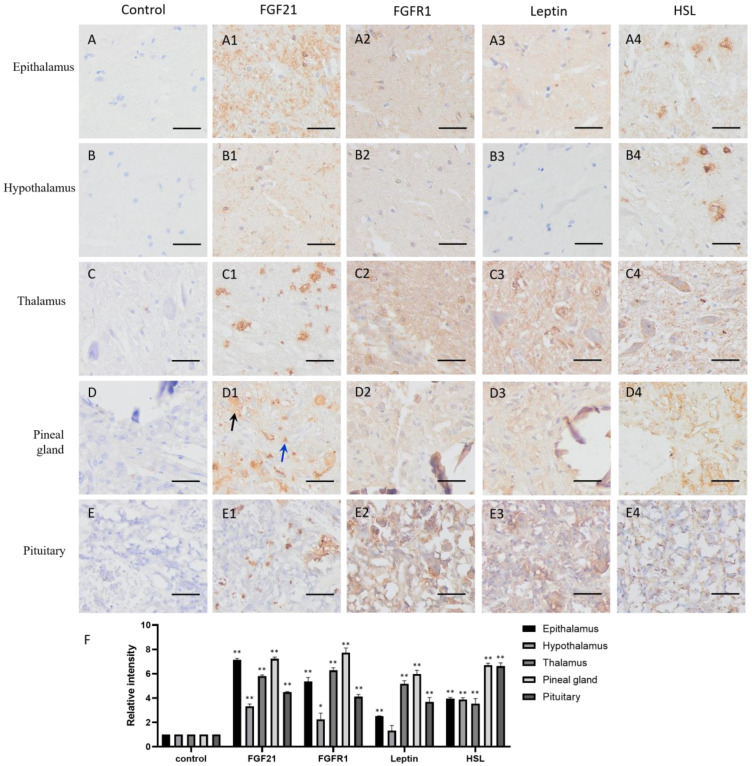
Distribution of FGF21, FGFR1, leptin, and hormone-sensitive lipase (HSL) in camel central nervous system (CNS) tissues. (**A**–**E**) In control group, primary antibodies were replaced by nonspecific IgG in an immunohistochemistry (IHC) assay; only the nuclei were stained blue with hematoxylin dye in camel epithalamus, hypothalamus, thalamus, pineal gland, and pituitary, respectively. (**A1**–**E1**) IHC staining of FGF21 in camel epithalamus, hypothalamus, thalamus, pineal gland, and pituitary, respectively. (**A2**–**E2**) IHC staining of FGFR1 in camel epithalamus, hypothalamus, thalamus, pineal gland, and pituitary, respectively. (**A3**–**E3**) IHC staining of leptin in camel epithalamus, hypothalamus, thalamus, pineal gland, and pituitary, respectively. (**A4**–**E4**) IHC staining of HSL in camel epithalamus, hypothalamus, thalamus, pineal gland, and pituitary, respectively. (**F**) The optical density (OD) of IHC stain was evaluated using Image-Pro Plus 6.0. Data are presented as mean ± SD of triplicate experiments. * *p* < 0.05, ** *p* < 0.01 (Student’s *t*-test). Scale bars: 50 μm.

FGF21 showed clear positive staining (yellow or brown) in the nervous system tissues compared with the negative controls (Figure 1(A1–E1,F)), including the epithalamus, hypothalamus, thalamus, pineal gland, and pituitary, indicating that nerve cells express and secrete FGF21 protein. Intriguingly, staining data showed that not all nerve tissue cells express FGF21, especially in the thalamus, pineal gland, and pituitary (Figure 1(C1–E1)). Neural tissue is composed of many different cell types and involved in the production of multiple hormones. This result demonstrated that nerve tissue can produce and secrete FGF21 in camels.

The IHC staining showed that the FGFR1 receptor is widely distributed in the CNS tissues and particularly in the thalamus and pituitary (Figure 1(A2–E2,F)), together with the fact that FGF21 exerts function mainly through a FGFR1-mediated pathway; the result indicated that nerve tissues are universally responsive to FGF21 signaling.

In addition, the lipid metabolism markers leptin and HSL are widely located in the CNS tissues and particularly in the thalamus and pituitary (Figure 1(A3–E3,A4–E4,F)). Interestingly, leptin and HSL showed the similar expression pattern with the FGFR1 receptor. They all showed high expression in the thalamus and pituitary but low expression in the epithalamus and hypothalamus (Figure 1(A3–E3,A4–E4,F)). It may be the parvocellular neurosecretory cells in the epithalamus and the magnocellular neurosecretory cells in the hypothalamus expressing leptin and HSL. The results suggested that fat metabolism is more active in the thalamus and pituitary than other CNS tissue, and active fat metabolism may be related to FGFR1 receptor-mediated signaling. Notably, the positive staining of HSL in the epithalamus and hypothalamus showed scattered distribution (Figure 1(A4,B4)), indicating that some specific cells in the epithalamus and hypothalamus are very active in lipolysis.

### 3.2. In Reproductive Tissue

**Figure 2:**
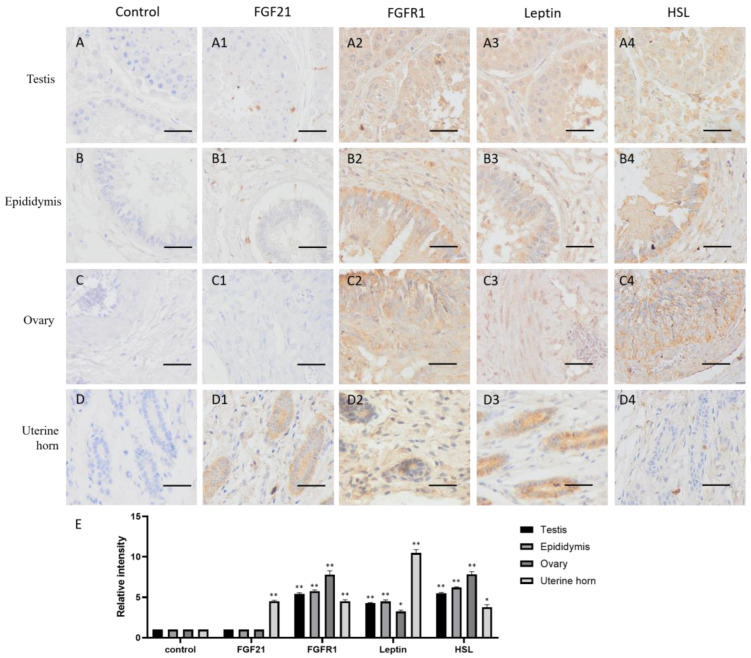
Distribution of FGF21, FGFR1, leptin, and hormone-sensitive lipase (HSL) in camel reproductive system tissues. (**A**–**D**) In control group, primary antibodies were replaced by nonspecific IgG in an immunohistochemistry (IHC) assay; only the nuclei were stained blue with hematoxylin dye in camel testis, epididymis, ovary, and uterine horn, respectively. (**A1**–**D1**) IHC staining of FGF21 in camel testis, epididymis, ovary, and uterine horn, respectively. (**A2**–**D2**) IHC staining of FGFR1 in camel testis, epididymis, ovary, and uterine horn, respectively. (**A3**–**D3**) IHC staining of leptin in camel testis, epididymis, ovary, and uterine horn, respectively. (**A4**–**D4**) IHC staining of HSL in camel testis, epididymis, ovary, and uterine horn, respectively. (**E**) The OD of IHC stain was evaluated using Image-Pro Plus 6.0. Data are presented as mean ± SD of triplicates. * *p* < 0.05, ** *p* < 0.01 (Student’s *t*-test). Scale bars: 50 μm.

FGF21 showed negative staining in most reproductive tissues, including the testis, epididymis and ovary (Figure 2(A2–C2)), but the uterine gland was deeply stained in the uterine horn (Figure 2(D2)). The result indicated that only the uterine gland expressed and secreted FGF21 protein in the camel reproductive system.

In contrast, the receptor FGFR1 showed widely positive staining (yellow or brown) in reproductive tissues, including the testis, epididymis, ovary, and uterine horn (Figure 2(A3–D3,E)), indicating that reproductive tissues are generally responsive to FGF21 signaling.

The lipid metabolism markers leptin and HSL are widely distributed in the testis, epididymis, and ovary and showed similar expression pattern with the FGFR1 receptor (Figure 2(A4–C4,E)), whereas in the uterine horn, leptin is only present in the uterine gland, and HSL is absent (Figure 2(D4)). These results indicated that in the reproductive system, fat metabolism is more active in gametogenic tissue including the testis, epididymis, and ovary, rather than uterine.

### 3.3. In Adipose and Muscle

**Figure 3:**
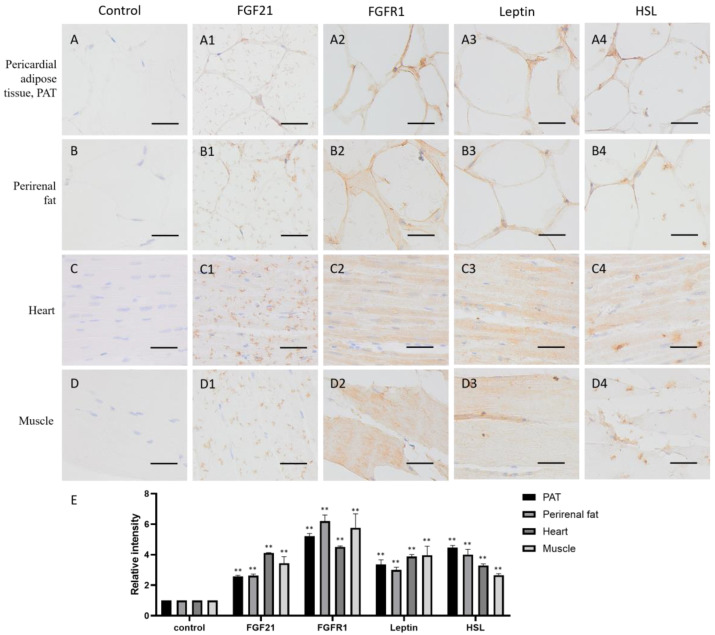
Distribution of FGF21, FGFR1, leptin, and hormone-sensitive lipase (HSL) in camel adipose and muscle tissues. (**A**–**D**) In control group, primary antibodies were replaced by nonspecific IgG in an immunohistochemistry (IHC) assay; only the nuclei were stained blue with hematoxylin dye in camel pericardial adipose tissue (PAT), perirenal fat, heart, and muscle, respectively. (**A1**–**D1**) IHC staining of FGF21 in camel PAT, perirenal fat, heart, and muscle, respectively. (**A2**–**D2**) IHC staining of FGFR1 in camel PAT, perirenal fat, heart, and muscle, respectively. (**A3**–**D3**) IHC staining of leptin in camel PAT, perirenal fat, heart, and muscle, respectively. (**A4**–**D4**) IHC staining of HSL in camel PAT, perirenal fat, heart, and muscle, respectively. (**E**) The OD of IHC stain was evaluated using Image-Pro Plus 6.0. Data are presented as mean ± SD of triplicates. ** *p* < 0.01 (Student’s *t*-test). Scale bars: 50 μm.

Adipose tissue plays an important role in energy storage. As expected, both in pericardial adipose tissue (PAT) and perirenal fat, FGF21, FGFR1, and the lipid metabolism markers leptin and HSL all are positive stained (yellow or brown) around the fat vacuoles (Figure 3(A1–A4,B1–B4,E)), whose formation is due to the dissolution of fat by organic solvents, such as xylene, during the specimen-making, indicating that adipose tissue not only produces FGF21 but also responds to FGF21 signaling, and lipid metabolism is very active.

Muscle is the contractile tissue found in animals, the function of which is to produce motion. FGF21 showed a scattered distribution in the muscle cells, including cardiac muscle and skeletal muscle (Figure 3(C1,D1)). In the heart, FGF21 is mainly localized in intercalated discs (Figure 3(C1)), which are complex structures that connect adjacent cardiac muscle cells [25]. In skeletal muscle, FGF21 is mainly localized in striations or linear marks (Figure 3(C1)), whereas FGFR1 and the lipid metabolism markers leptin and HSL are widely distributed in both cardiac and skeletal muscle tissue (Figure 3(C2–C4,D2–D4,E)). These data indicated that muscle tissue including cardiac and skeletal muscle are responsive to FGF21 signaling, and lipid metabolism is very active.

### 3.4. In Visceral Organs

**Figure 4:**
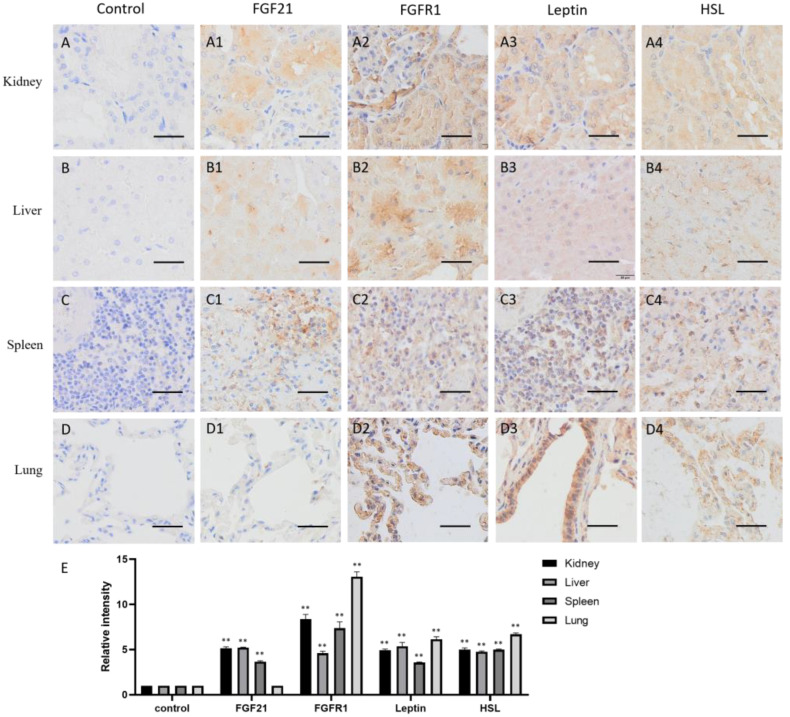
Distribution of FGF21, FGFR1, leptin, and hormone-sensitive lipase (HSL) in camel visceral organs. (**A**–**D**) In control group, primary antibodies were replaced by nonspecific IgG in an immunohistochemistry (IHC) assay; only the nuclei were stained blue with hematoxylin dye in camel kidney, liver, spleen, and lung, respectively. (**A1**–**D1**) IHC staining of FGF21 in camel kidney, liver, spleen, and lung, respectively. (**A2**–**D2**) IHC staining of FGFR1 in camel kidney, liver, spleen, and lung, respectively. (**A3**–**D3**) IHC staining of leptin in camel kidney, liver, spleen, and lung, respectively. (**A4**–**D4**) IHC staining of HSL in camel kidney, liver, spleen, and lung, respectively. (**E**) The OD of IHC stain was evaluated using Image-Pro Plus 6.0. Data are presented as mean ± SD of triplicates. ** *p* < 0.01 (Student’s *t*-test). Scale bars: 50 μm.

The distribution of FGF21 in visceral organs is different. In kidneys, FGF21 is mainly located in the proximal and distal convoluted canaliculi cells (Figure 4(A1)), suggesting that renal tubular epithelial cells express and secrete FGF21. FGFR1, leptin, and HSL are all highly and widely expressed in kidneys (Figure 4(A2–A4)), indicating that renal cells are responsive to FGF21 signaling, and lipid metabolism is active.

In the liver, FGF21, FGFR1, leptin, and HSL all are widely expressed (Figure 4(B1–B4)). Together with the fact that the liver is the major metabolic organ in the body, liver produces FGF21, responds to FGF21 signaling, and regulates lipid metabolism.

The spleen is one of the most important peripheral immune organs in animals and exerts multiple functions, including filtering for blood-borne pathogens and antigens, iron metabolism, erythrocyte homeostasis, and the generation of antigen-specific immune responses [26]. The spleen contains two main regions, white pulp and red pulp, and many kinds of cell populations. The IHC data showed that some cells in the red pulp were deeply stained by the FGF21 antibody (Figure 4(C1)), suggesting that some red pulp cells express FGF21 in the spleen. In contrast, almost all cells showed positive staining with the FGFR1, leptin, and HSL antibodies, indicating that FGFR1, leptin, and HSL are widely distributed in the spleen (Figure 4(C2–C4)). These results indicated that the spleen produces FGF21 and responds to FGF21 signaling and lipid metabolism.

The lungs are organs of the respiratory system that allow animals to take in and expel air. As shown in Figure 4D, the IHC staining showed that FGF21 is absent in alveolar epithelial cells (Figure 4(D1)). However, FGFR1, leptin, and HSL are highly expressed in the alveolar epithelial cells (Figure 4(D2–D4)). Intriguingly, leptin is highly expressed in the bronchial epithelial cells (Figure 4(D3)). These results indicated that bronchial epithelial cells do not produce FGF21 but may response to FGF21 signaling and lipid metabolism.

## 4. Discussion

As a hormone, FGF21 regulates glucose–lipid metabolism and is becoming a promising therapeutic target for metabolic disease. Camels have an efficient lipid reserve and metabolism and are able to survive long periods without feeding, which would be helpful to understand more about the fat conversion mode and provide an inspiration for developing a potential therapeutic method for human obesity. However, knowledge about FGF21-mediated lipid metabolism in camels is still limited.

The previous studies showed that FGF21 is widely expressed in metabolic organs, mainly produced by the liver and then by adipose tissue and skeletal muscle in humans and mice, and then enters the circulation to carry out its function [27,28]. However, our study found that in addition to metabolic organs, FGF21 also appeared in the camel central nerve tissue, spleen, kidney, and uterine gland but was absent in the lung and gametogenic tissue, including the testis, epididymis, and ovary (Figure 1, Figure 2, Figure 3 and Figure 4), which may be due to species diversity. In the spleen, only red pulp cells could express and secrete FGF21 (Figure 4). In striated muscle, including cardiac and skeletal muscle, FGF21 is only present at a fiber junction, such as an intercalated disk (Figure 3). Notably, neural tissue, including the hypothalamus, pineal gland, and pituitary, is composed of many different cell types and is involved in the production of multiple hormones, such as growth hormone (GH), adrenocorticotropin hormone (ACTH), luteinizing hormone (LH), follicle-stimulating hormone (FSH), prolactin (PRL), thyroid-stimulating hormone (TSH), and melatonin. Intriguingly, unlike humans and mice, in camels, FGF21 showed active expression in the nervous system, suggesting a special expression pattern of FGF21 in camels, which may be related to the adaptability of camels to arid environments and the specific regulation of lipid metabolism. Additionally, FGF21 may be another neuronal hormone in camels, which needs to be further confirmed in the future study.

As the main FGF21 receptor, FGFR1 mediates the activation of multiple downstream signaling pathways, including Ras/Raf MAPK, mTOR, and PI3K/AKT, although the signaling downstream is tissue-specific [10,16]. These signals involve biological functions such as triglyceride homeostasis, glucose uptake, amino acid transport, and energy expenditure [29]. Consistent with previous studies, this study also found that FGFR1 is expressed in almost all tissues and cells (Figure 1, Figure 2, Figure 3 and Figure 4). Notably, FGF21 acts through a cell-surface receptor complex comprised of FGFR1 and KLB; KLB is expressed in specific metabolic tissues thereby conferring signaling specificity for FGF21 [7,28], suggesting that identifying the tissue expression of KLB is more helpful to understand FGF21-induced signaling in camels.

To further explore the correlation between FGF21 and lipid metabolism in camels, this study also detected the distribution of the lipid metabolism markers leptin and HSL in camels. Leptin, encoded by the LEP gene, is an adipose-derived satiety hormone and exerts its physiological activity through a class I cytokine receptor, LEPR or Ob-R [30,31]. Leptin is considered as a mediator of the adaptation to fasting and an anti-obesity hormone [32], whose plasma levels correlate with fat stores and respond to changes in energy balance [33]. In addition, as a protein hormone, leptin also exerts potential functions in immune regulation and the pathogenesis of autoimmune diseases, such as the activation of monocytes, macrophages, neutrophils, and T lymphocytes [34,35]. HSL is a key intracellular neutral lipase in the regulation of lipolysis, which is a catabolic process leading to the breakdown of triacylglycerols stored in fat cells and the release of fatty acids and glycerol, also termed as fat mobilization [36].

Consistent with previous studies that leptin and HSL are mainly synthesized in adipose tissue but also expressed in the central nervous system and peripheral tissues [36,37], our study also confirmed that leptin and HSL are mainly located in metabolic and energy consuming organs, such as adipose tissue, muscles, the liver, kidney, spleen, lung, testis, epididymis, and ovary (Figure 2, Figure 3 and Figure 4), showing that leptin and HSL, as representations of lipid metabolism and energy supply, are highly expressed in tissue and organs with active metabolism. Intriguingly, in the CNS, leptin and HSL are mainly distributed in the epithalamus, thalamus, pineal gland, and pituitary but less in the hypothalamus (Figure 1), which is similar to the expression pattern of FGFR1, indicating that the activation of lipid metabolism may be related to FGFR1-mediated signaling. The cells expressing leptin and HSL in the epithalamus are parvocellular neurosecretory cells and magnocellular neurosecretory cells in the hypothalamus. Actually, leptin and HSL are excellent biomarkers for body fat content and related to disorders of glucose and lipid metabolism, such as insulin resistance, type-2 diabetes, and obesity [38,39]. Notably, leptin expression is extremely high in bronchial epithelial cells (Figure 4). The previous reports showed that leptin is key for fetal lung development and promoted the survival of alveolar epithelial cells [40,41]. Moreover, leptin is involved in immune responses via the activation of monocytes, macrophages, neutrophils, and T lymphocytes [34]. The high expression of leptin in camel bronchial epithelium is may be due to its role in the immune responses of respiratory mucosa, in addition to fat stores and energy balance, although the involved underlying mechanism is still to be determined by further investigations.

## 5. Conclusions

This study found that FGF21 was widely expressed in camel central nerve tissue and peripheral organs, including the hypothalamus, pineal gland, pituitary, adipose tissue, liver, kidney, spleen, heart, muscle, and uterine horn but was absent in the lung and gametogenic tissue, including the testis, epididymis, and ovary. The distribution of FGF21 in the hypothalamus, pineal gland, and pituitary demonstrated that FGF21 is another neuronal hormone related to lipid metabolism, glucose utilization, and energy homeostasis in camels. In striated muscle, FGF21 is only present at the fiber junction. FGFR1 is expressed in almost all tissues and cells, indicating that all tissues are responsive to FGF21 and other FGF-mediated signals. Leptin and HSL are mainly located in metabolic and energy consuming organs, and in the CNS, leptin and HSL showed a similar expression pattern with FGFR1. In addition, leptin expression is extremely high in camel bronchial epithelium which may be due to its role in the immune responses of respiratory mucosa, in addition to fat stores and energy balance. This study found a diversity of FGF21 expression patterns in camels, which will be helpful to understand more about the FGF21-mediated fat conversion mode and provide a reference for developing a potential therapeutic method for fat metabolism disease.

## Data Availability

Not applicable.
